# Supplementary material for: Skim Milk as a Multifunctional Cryoprotectant for Fish Probiotic Enterococcus spp.: Impact on Viability During Lyophilization and Long-Term Storage
Source: Microorganisms. 2025 Oct 30;13(11):2486. doi: 10.3390/microorganisms13112486 (PMC12654816; doi:10.3390/microorganisms13112486)
Supplement: Supplementary file 1 [file microorganisms-13-02486-s001.zip › microorganisms-3922020-Supplementary Figure S1.pdf]

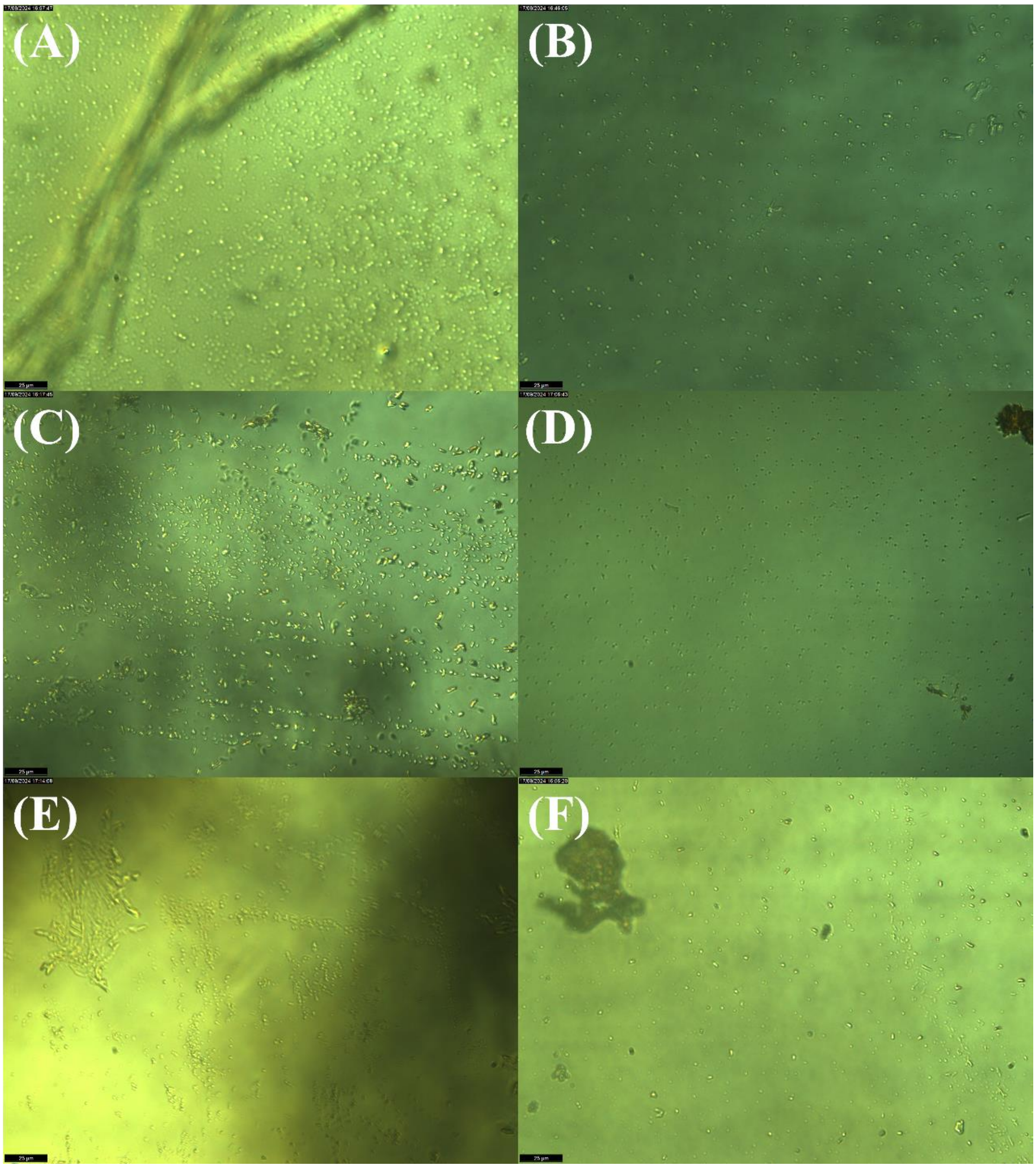

Figure S1: Photomicrographs of *E. faecium* CRBP46 cultures freeze-dried with different cryoprotectants and subjected to differential interference contrast microscopy. (A) maltodextrin; (B) skimmed milk; (C) trehalose; (D) sucrose; (E) fructose; (F) dextrose.
