# Supplementary material for: Skim Milk as a Multifunctional Cryoprotectant for Fish Probiotic Enterococcus spp.: Impact on Viability During Lyophilization and Long-Term Storage
Source: Microorganisms. 2025 Oct 30;13(11):2486. doi: 10.3390/microorganisms13112486 (PMC12654816; doi:10.3390/microorganisms13112486)
Supplement: Supplementary file 1 [file microorganisms-13-02486-s001.zip › microorganisms-3922020-Supplementary Tables S1 and S2.pdf]

Supplementary Table 1. Counts (log CFU/g) of *Enterococcus faecium* CRBP46 freeze-dried with different cryoprotectant agents after freeze-drying, and during storage at cooling (4 ± 0.6 ºC), room (25 ± 1.05 ºC) and freezing (-25 ± 1.1 ºC) temperatures.

	Storage (days) at 4°C			
**Cryoprotector**	**After freeze-drying**	**30**	**60**	**120**
CT	8.51 ± 0.07^{aB}	8.47 ± 0.14^{aD}	8.33 ± 0.16^{abD}	8.17 ± 0.0^{bC}
MD	9.10 ± 0.06^{aA}	8.87 ± 0.07^{aB}	8.91 ± 0.02^{aBC}	9.10 ± 0.10^{aAB}
SKM	9.13 ± 0.10^{aA}	9.14 ± 0.02^{aA}	9.14 ± 0.06^{aAB}	9.07 ± 0.06^{aAB}
TL	9.12 ± 0.13^{aA}	9.11 ± 0.02^{aA}	9.14 ± 0.09^{aAB}	8.87 ± 0.06^{bB}
SR	9.22 ± 0.07^{aA}	9.11 ± 0.05^{aA}	9.20 ± 0.07^{aA}	9.15 ± 0.02^{aA}
FT	9.19 ± 0.06^{aA}	8.90 ± 0.02^{bAB}	9.11 ± 0.05^{abAB}	9.14 ± 0.08^{abA}
DT	9.15 ± 0.03^{aA}	8.83 ± 0.13^{bBC}	8.76 ± 0.01^{bC}	9.23 ± 0.07^{aA}
	**Storage (days) at 25°C**			
**Cryoprotector**	**After freeze-drying**	**30**	**60**	**120**
CT	8.51 ± 0.07^{aB}	7.64 ± 0.12^{bB}	6.50 ± 0.01^{cC}	5.41 ± 0.06^{dC}
MD	9.10 ± 0.06^{aA}	9.17 ± 0.04^{aA}	9.15 ± 0.11^{aA}	7.93 ± 0.00^{bB}
SKM	9.13 ± 0.10^{aA}	9.18 ± 0.05^{aA}	8.58 ± 0.25^{bA}	9.04 ± 0.01^{aA}
TL	9.12 ± 0.13^{aA}	9.15 ± 0.05^{aA}	9.05 ± 0.03^{aA}	7.80 ± 0.38^{bB}
SR	9.22 ± 0.07^{aA}	9.07 ± 0.10^{aA}	8.47 ± 0.10^{bB}	8.65 ± 0.05^{bA}
FT	9.19 ± 0.06^{aA}	7.21 ± 0.12^{bB}	4.79 ± 0.13^{cD}	0.00 ± 0.00
DT	9.15 ± 0.03^{aA}	6.60 ± 0.52^{bC}	0.00 ± 0.00	0.00 ± 0.00
	**Storage (days) at -25 °C**			
**Cryoprotector**	**After freeze-drying**	**30**	**60**	**120**
CT	8.51 ± 0.07^{bB}	8.92 ± 0.10^{aB}	8.86 ± 0.07^{aC}	8.91 ± 0.05^{aB}
MD	9.10 ± 0.06^{bA}	9.24 ± 0.04^{abA}	9.34 ± 0.11^{aA}	9.14 ± 0.12^{abAB}
SKM	9.13 ± 0.10^{aA}	9.19 ± 0.10^{aA}	9.21 ± 0.02^{aAB}	9.33 ± 0.03^{aA}
TL	9.12 ± 0.13^{aA}	9.30 ± 0.04^{aA}	9.31 ± 0.04^{aAB}	9.20 ± 0.06^{aA}
SR	9.22 ± 0.07^{aA}	9.29 ± 0.06^{aA}	9.10 ± 0.07^{aB}	9.14 ± 0.05^{aAB}
FT	9.19 ± 0.06^{aA}	9.20 ± 0.01^{aA}	8.82 ± 0.14^{bC}	9.20 ± 0.00^{aA}
DT	9.15 ± 0.03^{aA}	9.18 ± 0.04^{aA}	9.20 ± 0.04^{aAB}	9.15 ± 0.10^{aA}

Note: $^{a-c}$ Superscript lowercase letters in the same row denote statistically different ($p < 0.05$) counts of cells after freeze-drying and during storage. $^{A-D}$ Superscript capital letters in the same column denote statistically different ($p < 0.05$) treatments of freeze-drying based on Tukey's test. The values represent the mean ± standard deviation obtained from independent samples (n=3). CT: control (phosphate-buffered saline - PBS); MD: maltodextrin; SKM: skimmed milk; TL: trehalose; SR: sucrose; FT: fructose; DT: dextrose.

Supplementary Table 2. Counts (Log CFU/g) of *Enterococcus gallinarum* CRBP19 freeze-dried with different cryoprotectant agents after freeze-drying, and during storage at cooling (4 ± 0.6ºC), room (25 ± 1.05ºC) and freezing (-25 ± 1.1 ºC) temperatures.

Cryoprotector	After freeze-drying	Storage (days) at 4ºC		
		30	60	120
CT	8.99 ± 0.04^{aB}	9.05 ± 0.08^{aAB}	8.69 ± 0.10^{bC}	8.98 ± 0.15^{aCD}
MD	9.22 ± 0.07^{aAB}	9.03 ± 0.02^{abB}	8.82 ± 0.14^{bcC}	8.70 ± 0.10^{cB}
SKM	9.29 ± 0.10^{aA}	9.12 ± 0.04^{aAB}	9.12 ± 0.04^{aB}	9.16 ± 0.11^{aBC}
TL	9.24 ± 0.04^{aAB}	9.31 ± 0.09^{aA}	9.18 ± 0.04^{aAB}	9.42 ± 0.03^{aAB}
SR	9.43 ± 0.03^{aA}	9.31 ± 0.08^{aA}	9.41 ± 0.03^{aA}	9.37 ± 0.04^{aAB}
FT	9.28 ± 0.06^{aA}	9.33 ± 0.02^{aA}	9.27 ± 0.22^{aAB}	9.51 ± 0.02^{aA}
DT	9.25 ± 0.18^{aAB}	9.26 ± 0.07^{aAB}	9.32 ± 0.08^{aAB}	9.41 ± 0.03^{aAB}
		**Storage (days) at 25ºC**		
**Cryoprotector**	**After freeze-drying**	**30**	**60**	**120**
CT	8.99 ± 0.04^{aB}	3.39 ± 0.14^{bE}	2.99 ± 0.09^{cD}	0.00 ± 0.00
MD	9.22 ± 0.07^{aAB}	3.74 ± 0.05^{bD}	2.76 ± 0.00^{cE}	0.00 ± 0.00
SKM	9.29 ± 0.10^{aA}	8.83 ± 0.05^{bA}	8.18 ± 0.04^{cA}	6.83 ± 0.03^{dA}
TL	9.24 ± 0.04^{aA}	5.97 ± 0.03^{bC}	5.16 ± 0.06^{cC}	3.98 ± 0.19^{dB}
SR	9.43 ± 0.03^{aA}	6.82 ± 0.02^{bB}	6.03 ± 0.03^{cB}	3.64 ± 0.45^{dB}
FT	9.28 ± 0.06^{A}	0.00 ± 0.00	0.00 ± 0.00	0.00 ± 0.00
DT	9.25 ± 0.18^{aA}	6.13 ± 0.05^{bC}	2.37 ± 0.08^{cF}	0.00 ± 0.00
		**Storage (days) at -25 ºC**		
**Cryoprotector**	**After freeze-drying**	**30**	**60**	**120**
CT	8.99 ± 0.04^{aB}	8.90 ± 0.09^{aB}	8.90 ± 0.07^{aB}	8.96 ± 0.12^{aB}
MD	9.22 ± 0.07^{aAB}	9.34 ± 0.11^{aA}	9.24 ± 0.11^{aA}	9.29 ± 0.11^{aA}
SKM	9.29 ± 0.10^{aA}	9.26 ± 0.04^{aA}	9.26 ± 0.06^{aA}	9.20 ± 0.07^{aAB}
TL	9.24 ± 0.04^{aAB}	9.32 ± 0.01^{aA}	9.34 ± 0.19^{aA}	9.31 ± 0.03^{aA}
SR	9.43 ± 0.03^{aA}	9.35 ± 0.10^{aA}	9.34 ± 0.04^{aA}	9.40 ± 0.05^{aA}
FT	9.28 ± 0.06^{aA}	9.39 ± 0.03^{aA}	9.28 ± 0.01^{aA}	9.42 ± 0.02^{aA}
DT	9.25 ± 0.18^{aAB}	9.35 ± 0.01^{aA}	9.27 ± 0.10^{aA}	9.31 ± 0.08^{aA}

Note: $^{a\text{-}c}$ Superscript lowercase letters in the same row denote statistically different ($p < 0.05$) counts of cells after freeze-drying and during storage. $^{A\text{-}F}$ Superscript capital letters in the same column denote statistically different ($p < 0.05$) treatments of freeze-drying based on Tukey's test. The values represent the mean ± standard deviation obtained from independent samples (*n*=3). CT: control (phosphate-buffered saline - PBS); MD: maltodextrin; SKM: skimmed milk; TL: trehalose; SR: sucrose; FT: fructose; DT: dextrose.
