# Supplementary material for: Skim Milk as a Multifunctional Cryoprotectant for Fish Probiotic Enterococcus spp.: Impact on Viability During Lyophilization and Long-Term Storage
Source: Microorganisms. 2025 Oct 30;13(11):2486. doi: 10.3390/microorganisms13112486 (PMC12654816; doi:10.3390/microorganisms13112486)
Supplement: Supplementary file 1 [file microorganisms-13-02486-s001.zip › microorganisms-3922020-Supplementary Tables S3 and S4.pdf]

Supplementary Table 3. Viable cell counts (log CFU/g) of *Enterococcus faecium* CRBP46 freeze-dried with different cryoprotectant agents during exposure to pH values.

Cryoprotector	0 min	120 min	240 min	Survival rate (%)
**pH (3.0)**				
**Cryoprotector**	**0 min**	**120 min**	**240 min**	**Survival rate (%)**
CT	7.23 ± 0.08^{aD}	7.35 ± 0.03^{aC}	7.40 ± 0.03^{aC}	102.37 ± 0.91^{A}
MD	8.79 ± 0.05^{bC}	-	9.01 ± 0.03^{aA}	102.60 ± 0.90^{A}
SKM	9.13 ± 0.02^{aAB}	9.07 ± 0.04^{aA}	9.06 ± 0.06^{aA}	99.28 ± 0.39^{B}
TL	9.30 ± 0.07^{aA}	9.24 ± 0.06^{abA}	9.09 ± 0.02^{bA}	97.81 ± 0.68^{B}
SR	9.15 ± 0.10^{aAB}	9.18 ± 0.06^{aA}	9.09 ± 0.03^{aA}	99.36 ± 1.08^{B}
FT	9.13 ± 0.12^{aAB}	8.50 ± 0.01^{bB}	8.38 ± 0.02^{bB}	91.77 ± 1.44^{C}
DT	9.08 ± 0.07^{aB}	9.09 ± 0.04^{aA}	8.93 ± 0.05^{aA}	98.37 ± 1.31^{B}
**pH (5.0)**				
**Cryoprotector**	**0 min**	**120 min**	**240 min**	**Survival rate (%)**
CT	8.14 ± 0.09^{bC}	8.62 ± 0.08^{aB}	8.64 ± 0.11^{aC}	106.11 ± 0.87^{A}
MD	9.20 ± 0.04^{aAB}	9.16 ± 0.10^{aA}	9.18 ± 0.07^{aAB}	99.79 ± 0.88^{B}
SKM	9.19 ± 0.14^{aAB}	9.12 ± 0.06^{aA}	9.06 ± 0.01^{aB}	98.63 ± 1.38^{B}
TL	9.24 ± 0.08^{aAB}	9.15 ± 0.13^{aA}	9.30 ± 0.08^{aAB}	100.59 ± 0.14^{B}
SR	9.29 ± 0.06^{aAB}	9.15 ± 0.04^{aA}	9.19 ± 0.04^{aAB}	99.00 ± 2.04^{B}
FT	9.08 ± 0.05^{aB}	9.14 ± 0.10^{aA}	9.16 ± 0.01^{aAB}	100.87 ± 0.41^{B}
DT	9.34 ± 0.04^{aA}	9.27 ± 0.03^{aA}	9.33 ± 0.07^{aA}	99.82 ± 0.83^{B}
**pH (7.0)**				
**Cryoprotector**	**0 min**	**120 min**	**240 min**	**Survival rate (%)**
CT	8.45 ± 0.19^{aC}	8.47 ± 0.02^{aD}	8.31 ± 0.10^{aD}	98.37 ± 1.07^{AB}
MD	9.12 ± 0.10^{aB}	8.86 ± 0.07^{bC}	9.14 ± 0.01^{aC}	100.23 ± 0.99^{A}
SKM	9.10 ± 0.10^{aB}	9.00 ± 0.02^{aBC}	8.94 ± 0.02^{aBC}	98.24 ± 0.90^{AB}
TL	9.10 ± 0.00^{aB}	9.19 ± 0.09^{aAB}	9.08 ± 0.02^{aAB}	99.71 ± 0.14^{AB}
SR	9.44 ± 0.11^{aA}	9.27 ± 0.10^{aA}	9.31 ± 0.01^{aA}	98.62 ± 1.11^{AB}
FT	9.43 ± 0.03^{aA}	9.27 ± 0.05^{abA}	9.18 ± 0.01^{bA}	97.41 ± 0.18^{B}
DT	9.21 ± 0.09^{aAB}	9.10 ± 0.09^{aAB}	9.05 ± 0.03^{aAB}	98.76 ± 0.76^{AB}

Note: $^{a-b}$ Superscript lowercase letters in the same row denote statistically different ($p < 0.05$) counts of cells during exposure to different pH values. $^{A-D}$ Superscript capital letters in the same column denote statistically different ($p < 0.05$) treatments, based on Tukey's test. The values represent the mean ± standard deviation obtained from independent samples (n=3). CT: control (phosphate-buffered saline - PBS); MD: maltodextrin; SKM: skimmed milk; TL: trehalose; SR: sucrose; FT: fructose; DT: dextrose.

Supplementary Table 4. Viable cell counts (log CFU/g) of *Enterococcus gallinarum* CRBP19 freeze-dried with different cryoprotectant agents during exposure to pH values.

		**pH (3.0)**		
**Cryoprotector**	**0 min**	**120 min**	**240 min**	**Survival rate (%)**
CT	7.49 ± 0.26^{aD}	7.38 ± 0.17^{aD}	7.48 ± 0.00^{aC}	99.32 ± 3.49^{AB}
MD	8.28 ± 0.04^{aC}	8.13 ± 0.03^{aC}	8.07 ± 0.11^{aB}	97.42 ± 1.07^{AB}
SKM	9.35 ± 0.10^{aAB}	9.24 ± 0.07^{aB}	9.14 ± 0.07^{aA}	97.79 ± 0.64^{B}
TL	9.24 ± 0.01^{aB}	9.29 ± 0.02^{aAB}	9.36 ± 0.04^{aA}	101.30 ± 0.50^{A}
SR	9.57 ± 0.01^{aA}	9.56 ± 0.03^{aA}	9.34 ± 0.09^{aA}	97.65 ± 0.88^{B}
FT	9.52 ± 0.02^{aAB}	9.44 ± 0.06^{aAB}	9.28 ± 0.14^{aA}	97.43 ± 1.41^{AB}
DT	9.44 ± 0.01^{aAB}	9.32 ± 0.03^{aAB}	9.18 ± 0.03^{aA}	97.19 ± 0.23^{B}
		**pH (5.0)**		
**Cryoprotector**	**0 min**	**120 min**	**240 min**	**Survival rate (%)**
CT	7.57 ± 0.03^{aD}	7.33 ± 0.08^{aD}	7.52 ± 0.10^{aD}	99.27 ± 1.69^{AB}
MD	8.49 ± 0.01^{aC}	8.05 ± 0.12^{bC}	8.10 ± 0.10^{bC}	95.38 ± 1.18^{BC}
SKM	9.23 ± 0.09^{aB}	8.86 ± 0.10^{bB}	8.91 ± 0.10^{bB}	96.47 ± 1.83^{BC}
TL	9.30 ± 0.12^{aAB}	9.26 ± 0.11^{aA}	9.33 ± 0.11^{aA}	100.33 ± 1.62^{A}
SR	9.56 ± 0.06^{aA}	9.44 ± 0.05^{aA}	9.38 ± 0.17^{aA}	98.06 ± 1.28^{AB}
FT	9.58 ± 0.09^{aA}	9.36 ± 0.07^{aA}	9.10 ± 0.13^{bAB}	95.04 ± 0.52^{C}
DT	9.34 ± 0.11^{aAB}	9.34 ± 0.06^{aA}	9.12 ± 0.05^{aAB}	97.59 ± 1.33^{AB}
		**pH (7.0)**		
**Cryoprotector**	**0 min**	**120 min**	**240 min**	**Survival rate (%)**
CT	7.54 ± 0.22^{aD}	7.53 ± 0.07^{aD}	7.60 ± 0.09^{aD}	100.79 ± 3.63^{A}
MD	8.45 ± 0.09^{aC}	8.16 ± 0.07^{bC}	8.04 ± 0.02^{bC}	95.15 ± 0.72^{B}
SKM	8.81 ± 0.06^{aB}	8.82 ± 0.03^{aB}	8.70 ± 0.06^{aB}	98.70 ± 0.07^{AB}
TL	9.48 ± 0.08^{aA}	9.24 ± 0.06^{aA}	9.25 ± 0.01^{aA}	97.64 ± 0.79^{AB}
SR	9.47 ± 0.05^{aA}	9.46 ± 0.02^{aA}	9.05 ± 0.17^{bA}	95.61 ± 1.27^{B}
FT	9.44 ± 0.07^{aA}	9.35 ± 0.02^{aA}	9.08 ± 0.05^{bA}	96.16 ± 1.18^{B}
DT	9.51 ± 0.02^{aA}	9.30 ± 0.02^{abA}	9.14 ± 0.04^{aA}	96.09 ± 0.64^{B}

Note: $^{a\text{-}b}$ Superscript lowercase letters in the same row denote statistically different ($p < 0.05$) counts of cells during exposure to different pH values. $^{A\text{-}D}$ Superscript capital letters in the same column denote statistically different ($p < 0.05$) treatments, based on Tukey's test. The values represent the mean ± standard deviation obtained from independent samples (n=3). CT: control (phosphate-buffered saline - PBS); MD: maltodextrin; SKM: skimmed milk; TL: trehalose; SR: sucrose; FT: fructose; DT: dextrose.
