# Supplementary material for: Skim Milk as a Multifunctional Cryoprotectant for Fish Probiotic Enterococcus spp.: Impact on Viability During Lyophilization and Long-Term Storage
Source: Microorganisms. 2025 Oct 30;13(11):2486. doi: 10.3390/microorganisms13112486 (PMC12654816; doi:10.3390/microorganisms13112486)
Supplement: Supplementary file 1 [file microorganisms-13-02486-s001.zip › microorganisms-3922020-Supplementary Tables S5 and S6.pdf]

Supplementary Table 5. Viable cell counts (log CFU/g) of *Enterococcus faecium* CRBP46 freeze-dried with different cryoprotectant agents during exposure to bile salts.

			**Bile salts (0.0%)**		
**Cryoprotector**	**0 min**	**60 min**	**180 min**	**300 min**	**Survival rate (%)**
CT	8.65 ± 0.05^{aC}	8.71 ± 0.03^{aB}	8.69 ± 0.08^{aB}	8.70 ± 0.04^{aD}	100.60 ± 0.78^{AB}
MD	9.23 ± 0.03^{aA}	9.32 ± 0.04^{aA}	9.25 ± 0.09^{aA}	9.24 ± 0.13^{aA}	100.10 ± 1.71^{AB}
SKM	9.31 ± 0.09^{abA}	9.30 ± 0.03^{abA}	9.36 ± 0.10^{aA}	9.12 ± 0.02^{bC}	97.95 ± 0.86^{B}
TL	9.19 ± 0.08^{aA}	9.32 ± 0.04^{aA}	9.32 ± 0.03^{aA}	9.17 ± 0.09^{aBC}	99.77 ± 1.42^{AB}
SR	9.25 ± 0.11^{aA}	9.26 ± 0.11^{aA}	9.39 ± 0.07^{aA}	9.37 ± 0.04^{aAB}	101.28 ± 1.26^{A}
FT	9.37 ± 0.04^{aA}	9.43 ± 0.11^{aA}	9.31 ± 0.18^{aA}	9.46 ± 0.02^{aA}	100.96 ± 0.71^{AB}
DT	8.99 ± 0.10^{bB}	9.28 ± 0.09^{aA}	9.27 ± 0.04^{aA}	9.06 ± 0.03^{abBC}	100.84 ± 0.79^{AB}
			**Bile salts (0.4%)**		
**Cryoprotector**	**0 min**	**60 min**	**180 min**	**300 min**	**Survival rate (%)**
CT	6.97 ± 0.16^{aC}	7.16 ± 0.05^{aC}	7.06 ± 0.13^{aE}	7.18 ± 0.02^{aD}	102.93 ± 2.17^{A}
MD	8.50 ± 0.02^{aB}	8.09 ± 0.28^{abB}	8.25 ± 0.04^{abBC}	8.04 ± 0.05^{bC}	94.56 ± 0.55^{B}
SKM	9.03 ± 0.04^{aA}	9.09 ± 0.09^{aA}	9.10 ± 0.02^{aA}	8.99 ± 0.07^{aA}	99.55 ± 0.79^{A}
TL	8.59 ± 0.19^{aB}	8.37 ± 0.12^{abB}	8.15 ± 0.13^{bC}	8.17 ± 0.03^{abBC}	95.08 ± 1.87^{B}
SR	8.44 ± 0.17^{aB}	8.28 ± 0.07^{aB}	8.59 ± 0.12^{aB}	8.49 ± 0.09^{aB}	100.59 ± 1.26^{A}
FT	8.78 ± 0.13^{aAB}	7.26 ± 0.19^{bC}	7.14 ± 0.11^{bE}	7.17 ± 0.13^{bD}	81.60 ± 0.62^{C}
DT	8.78 ± 0.06^{aAB}	7.95 ± 0.27^{bB}	7.78 ± 0.19^{bcD}	7.50 ± 0.19^{cD}	85.43 ± 1.57^{C}
			**Bile salts (0.7%)**		
**Cryoprotector**	**0 min**	**60 min**	**180 min**	**300 min**	**Survival rate (%)**
CT	6.53 ± 0.02^{aD}	6.54 ± 0.17^{aC}	6.75 ± 0.07^{abD}	7.18 ± 0.04^{aD}	109.87 ± 0.51^{A}
MD	8.27 ± 0.06^{aBC}	8.00 ± 0.17^{abB}	7.86 ± 0.13^{abBC}	7.55 ± 0.09^{bCD}	91.30 ± 0.70^{D}
SKM	8.85 ± 0.10^{aA}	8.89 ± 0.13^{aA}	9.12 ± 0.05^{aA}	9.14 ± 0.01^{aA}	103.37 ± 1.07^{B}
TL	8.03 ± 0.20^{aC}	8.06 ± 0.41^{aB}	8.21 ± 0.07^{aB}	7.92 ± 0.15^{aBC}	98.65 ± 0.59^{C}
SR	8.39 ± 0.05^{abBC}	8.64 ± 0.14^{aA}	8.23 ± 0.19^{abB}	8.16 ± 0.13^{bB}	97.21 ± 1.80^{C}
FT	8.28 ± 0.04^{aBC}	7.77 ± 0.03^{bB}	7.57 ± 0.06^{bC}	7.72 ± 0.08^{bBC}	93.31 ± 1.34^{D}
DT	8.55 ± 0.26^{aAB}	8.12 ± 0.14^{abB}	$8.14 \pm 0.10a^{bB}$	7.93 ± 0.06^{bBC}	92.79 ± 2.31^{D}
			**Bile salts (1.0%)**		
**Cryoprotector**	**0 min**	**60 min**	**180 min**	**300 min**	**Survival rate (%)**
CT	6.80 ± 0.09^{aE}	6.83 ± 0.04^{aD}	6.92 ± 0.03^{aE}	6.96 ± 0.02^{aE}	102.38 ± 1.54^{A}
MD	8.48 ± 0.12^{aCD}	8.29 ± 0.08^{abC}	8.06 ± 0.03^{bC}	8.09 ± 0.01^{bD}	95.38 ± 1.29^{BC}
SKM	9.17 ± 0.03^{aA}	9.13 ± 0.10^{aA}	9.04 ± 0.03^{aA}	9.09 ± 0.05^{aA}	99.12 ± 0.68^{AB}
TL	8.23 ± 0.08^{aD}	8.35 ± 0.00^{aBC}	7.88 ± 0.09^{bCD}	8.22 ± 0.02^{aB}	99.87 ± 1.19^{A}
SR	8.99 ± 0.03^{aAB}	8.66 ± 0.11^{bB}	8.44 ± 0.08^{bcB}	8.14 ± 0.11^{cB}	90.51 ± 1.09^{D}
FT	8.68 ± 0.05^{aBC}	8.10 ± 0.13^{bC}	7.72 ± 0.21^{cD}	7.71 ± 0.17^{cC}	88.90 ± 2.49^{D}
DT	8.64 ± 0.20^{aC}	8.36 ± 0.18^{aBC}	7.96 ± 0.18^{bCD}	7.91 ± 0.02^{bBC}	91.51 ± 1.91^{CD}

Note: $^{a\text{-}c}$ Superscript lowercase letters in the same row denote statistically different ($p < 0.05$) counts of cells during exposure to different concentrations of bile salts. $^{A\text{-}E}$ Superscript capital letters in the same column denote statistically different ($p < 0.05$) treatments, based on Tukey's test. The values represent the mean ± standard deviation obtained from independent samples (n=3). CT: control (phosphate-buffered saline - PBS); MD: maltodextrin; SKM: skimmed milk; TL: trehalose; SR: sucrose; FT: fructose; DT: dextrose.

Supplementary Table 6. Viable cell counts (log CFU/g) of *Enterococcus gallinarum* CRBP19 freeze-dried with different cryoprotectant agents during exposure to bile salts.

			**Bile salts (0.0%)**		
**Cryoprotector**	**0 min**	**60 min**	**180 min**	**300 min**	**Survival rate (%)**
CT	8.51 ± 0.02^{bB}	8.78 ± 0.11^{aB}	8.64 ± 0.03^{abB}	8.52 ± 0.11^{bB}	100.08 ± 1.04^{A}
MD	8.43 ± 0.06^{aB}	8.44 ± 0.03^{aC}	7.96 ± 0.02^{bC}	8.08 ± 0.03^{bC}	95.90 ± 0.79^{B}
SKM	9.27 ± 0.06^{aA}	9.14 ± 0.08^{aA}	9.08 ± 0.04^{aA}	9.09 ± 0.04^{aA}	98.10 ± 1.07^{AB}
TL	9.17 ± 0.07^{aA}	9.13 ± 0.07^{aA}	9.15 ± 0.07^{aA}	9.10 ± 0.09^{aA}	99.22 ± 0.66^{A}
SR	9.20 ± 0.03^{aA}	9.28 ± 0.02^{aA}	9.22 ± 0.08^{aA}	9.21 ± 0.12^{aA}	100.10 ± 1.63^{A}
FT	9.33 ± 0.08^{aA}	9.30 ± 0.06^{abA}	9.16 ± 0.11^{abA}	9.10 ± 0.02^{bA}	97.53 ± 0.62^{AB}
DT	9.19 ± 0.04^{abA}	9.30 ± 0.02^{aA}	9.02 ± 0.01^{bA}	9.04 ± 0.02^{bA}	98.40 ± 0.52^{AB}
			**Bile salts (0.4%)**		
**Cryoprotector**	**0 min**	**60 min**	**180 min**	**300 min**	**Survival rate (%)**
CT	7.52 ± 0.10^{aD}	6.81 ± 0.01^{bD}	6.70 ± 0.09^{bE}	-	89.17 ± 0.29^{*B}
MD	7.82 ± 0.04^{aC}	7.08 ± 0.05^{bD}	6.94 ± 0.05^{bDE}	-	88.80 ± 0.17^{*B}
SKM	8.94 ± 0.03^{aA}	8.86 ± 0.07^{aA}	9.00 ± 0.04^{aA}	-	100.69 ± 0.18^{*A}
TL	8.86 ± 0.06^{aA}	7.72 ± 0.09^{bC}	7.35 ± 0.05^{cC}	-	83.01 ± 0.04^{*C}
SR	8.88 ± 0.08^{aA}	8.39 ± 0.04^{bB}	7.87 ± 0.08^{cB}	-	88.57 ± 0.58^{*B}
FT	8.51 ± 0.08^{aB}	7.49 ± 0.04^{bC}	7.16 ± 0.11^{cCD}	-	84.15 ± 0.90^{*C}
DT	8.91 ± 0.07^{aA}	7.67 ± 0.09^{bC}	7.13 ± 0.27^{cCD}	-	80.01 ± 2.48^{*D}
			**Bile salts (0.7%)**		
**Cryoprotector**	**0 min**	**60 min**	**180 min**	**300 min**	**Survival rate (%)**
CT	5.66 ± 0.17^{aE}	5.60 ± 0.02^{aF}	5.66 ± 0.06^{aD}	-	100.06 ± 3.60^{*A}
MD	7.67 ± 0.00^{aD}	5.98 ± 0.21^{bE}	5.66 ± 0.10^{cD}	-	73.83 ± 1.33^{*C}
SKM	8.86 ± 0.11^{aA}	9.03 ± 0.05^{aA}	8.91 ± 0.10^{aA}	8.84 ± 0.09^{aA}	99.80 ± 1.26^{A}
TL	8.32 ± 0.19^{aBC}	8.17 ± 0.09^{aC}	8.20 ± 0.13^{aB}	8.07 ± 0.02^{aB}	96.97 ±2.36^{A}
SR	8.22 ± 0.07^{abB}	8.52 ± 0.11^{aB}	8.09 ± 0.09^{bBC}	8.07 ±0.04^{bB}	98.17 ± 1.35^{A}
FT	8.55 ± 0.07^{aAB}	7.94 ± 0.04^{bC}	7.82 ± 0.14^{bC}	7.74 ± 0.13^{bC}	90.56 ± 2.24^{B}
DT	8.23 ± 0.01^{aC}	7.60 ± 0.08^{cD}	7.95 ± 0.06^{abBC}	7.76 ± 0.11^{bcBC}	94.29 ± 1.37^{A}
			**Bile salts (1.0%)**		
**Cryoprotector**	**0 min**	**60 min**	**180 min**	**300 min**	**Survival rate (%)**
CT	6.23 ± 0.15^{bD}	6.32 ± 0.22^{bE}	6.82 ± 0.08^{aE}	6.58 ± 0.14^{abD}	105.76 ± 4.34^{A}
MD	7.59 ± 0.06^{aC}	6.84 ± 0.04^{bD}	6.35 ± 0.17^{cD}	-	83.61 ± 0.22^{*C}
SKM	8.85 ± 0.06^{aA}	8.91 ± 0.06^{aA}	8.84 ± 0.05^{aA}	8.97 ± 0.00^{aA}	101.29 ± 0.90^{A}
TL	8.41 ± 0.06^{aB}	8.23 ± 0.05^{aB}	8.27 ± 0.02^{aB}	8.17 ± 0.09^{aB}	97.16 ± 0.50^{A}
SR	8.49 ± 0.19^{aAB}	8.20 ± 0.05^{abB}	8.00 ± 0.04^{bBC}	7.95 ± 0.05^{bB}	93.60 ± 1.53^{B}
FT	8.38 ± 0.16^{aB}	7.65 ± 0.21^{bC}	7.64 ± 0.22^{bC}	7.53 ± 0.21^{bC}	89.98 ± 5.97^{B}
DT	8.16 ± 0.07^{aB}	7.40 ± 0.05^{cC}	7.77 ± 0.06^{bC}	7.79 ± 0.08^{abBC}	95.57 ± 0.16^{BC}

Note: $^{a-c}$ Superscript lowercase letters in the same row denote statistically different ($p < 0.05$) counts of cells during exposure to different concentrations of bile salts. $^{A-E}$ Superscript capital letters in the same column denote statistically different ($p < 0.05$) treatments, based on Tukey's test. The values represent the mean ± standard deviation obtained from independent samples (n=3). CT: control (phosphate-buffered saline - PBS); MD: maltodextrin; SKM: skimmed milk; TL: trehalose; SR: sucrose; FT: fructose; DT: dextrose. *The survival rate of *E. gallinarum* CRBP19 was determined after 180 minutes of exposure to bile salts.
